# An investigation of the corrosion behavior of zinc-coated stainless steel orthodontic wires: the effect of physical vapor deposition method

**DOI:** 10.1186/s12903-024-04242-5

**Published:** 2024-05-09

**Authors:** Maryam Karandish, Negar Hajipour, Hanieh Yazdani, Mona Mahdavi, Mansour Rahsepar

**Affiliations:** 1 Orthodontic Department, Dental School, Shiraz University of Medical Sciences, Shiraz, Iran; 2 Student Research Center, Dental School, Shiraz University of Medical Sciences, Shiraz, Iran; 3 Department of Materials Science and Engineering, School of Engineering, Shiraz University, Shiraz, Iran

**Keywords:** Corrosion, Stainless steel, Zinc, Coated materials, Biocompatible, Orthodontic wires

## Abstract

**Background:**

Releasing of metal ions might implicate in allergic reaction as a negative subsequent of the corrosion of Stainless Steel (SS304) orthodontic wires. The aim of this study was to evaluate the corrosion resistance of zinc-coated (Zn-coated) SS orthodontic wires.

**Methods:**

Zinc coating was applied on SS wires by PVD method. Electrochemical impedance spectroscopy (EIS), Potentiodynamic polarization tests and Tafel analysis methods were used to predict the corrosion behavior of Zn-coated and uncoated SS wires in both neutral and acidic environments.

**Results:**

The values of E_corr_ ,i_corr_ and R_ct ,_which were the electrochemical corrosion characteristics, reported better corrosion behavior of Zn-coated SS wires against uncoated ones in both artificial saliva and fluoride-containing environments. Experimental results of the Tafel plot analyses were consistent with that of electrochemical impedance spectroscopy analyses for both biological solutions.

**Conclusion:**

Applying Zn coating on bare SS orthodontic wire by PVD method might increase the corrosion resistance of the underlying stainless-steel substrate.

## Introduction

Stainless steel (SS) has been introduced around 1930 as an alloy for the fabrication of orthodontic arch wires and is still a broadly used appliance in orthodontic practice [1]. The corrosion resistance of SS wires are relatively favorable. However, this material is challenged by the hostile environment in the mouth, as it is susceptible to localized corrosion in a low pH environment containing chloride and fluoride ions [2, 3]. Previous researches showed high concentrations of nickel in the saliva and oral mucosa of patients wearing orthodontic appliances [4]. To minimize the risk of hypersensitivity reactions from nickel, the corrosion resistance of the stainless steel should be maximized to control the nickel ion release from the alloy. Coating is a logical way to enhance surface-dominated properties, like resistance to corrosion, ion release or wear, without compromising the mechanical properties of the bulk [5]. PVD technology lays a thin, uniform film layer on the surface of various materials [6]. This process consists of atomic deposition procedures in which a material is vaporized from solid or liquid sources in the form of atoms or molecules and transported in the form of vapor through a vacuum or low-pressure gaseous (plasma) environment to a substrate, where it finally condenses [7]. Among the metallic oxides, the zinc oxide (ZnO) has received considerable attention in producing anti-corrosion coating layers [8]. ZnO is a new multifunctional ion in organic materials, which has stable physical and chemical properties, high oxidation activity, ease of synthesis, and widely potential applications in many research areas [9].

Kachoei et al. [10] depositioned ZnO nano-particles on ss orthodontic wires by chemical solution method for friction reduction puropose. Also, Masahiro Goto et al. [11] evaluated reduction in the frictional force of Zno coatings on SS substrates which were deposited by Rf magnetron sputtering. Gopi et al. [12] have coated the surface of surgical grade SS with hydroxyapatite (HAp) by electro-deposition method and reported that the coating could enhance the longevity of the alloy in Ringer’s solution. Tavares et al. [13] applied ethylene glycol plasma polymer-coated titanium nanoparticles on 316 L SS surface and observed an increment of hydrophilicity and general corrosion resistance of the surface. Muthukumaran et al. [14] investigated the corrosion and hardness of ion implanted AISI 316 L stainless steel. TiN-ions implantation on SS 316 L has been investigated by Omrani et al. They concluded that corrosion currents decreased, and corrosion resistance improved in TiN implanted samples compared with that of the bare SSI316L [15]. Kumar et al. coated the SS specimen (304 L SS) by depositing nanoparticles of ZnO in 2018. Electrochemical Impedance Spectroscopy and Tafel plot parameters of the electrochemical corrosion tests indicated that the anticorrosion properties of the coated thin films were substantially higher than that of bare steel [16]. Ming Liu et al. [17] investigated corrosion and passive film characteristics of 3D-printed NiTi shape memory alloys in artificial saliva. They concluded that the L-PBF NiTi alloy prepared at a linear energy density of 0.2 J·m^− 1^ and volumetric energy density of 56 J·mm^− 3^ shows the least defects and best corrosion protection.

Comprehensive orthodontic treatment increases the susceptibility to enamel demineralization due to unique environment for colonization of microorganism [18]. On the other hand, orthodontists commonly prescribe a daily topical fluoride for prevention of enamel demineralization during treatment. It has been declared that commercially available topical fluoride prophylactic agents may exacerbate corrosive interaction of SS wires [19]. It is well known that SS is prone to pitting and crevice corrosion in solutions containing chloride ions [20]. The corrosion rate could be evaluated by weight loss test and chemical analysis of the metallic ions in bulk, but the best known technique for evaluation of the instantaneous corrosion rate in-vitro and in the field is the Polarization Resistance methods due to their high sensitivity [21].

Due to scarce studies on coating SS orthodontic wires with environmentally friendly Zn particles, the purpose of this study is to discover the corrosion protection behavior of Zn coated stainless steel orthodontic wires in both distilled water and fluoride contain medium.

## Materials and methods

Four 0.019 × 0.025 straight SS orthodontics wires (G&H, USA) [SS304 - AISI304 which corresponds to Z7CN18-09 according to the AFNOR standard.] with a length of 5 cm were used in this study. The specimens were divided into two groups of Zn-coated (case) and uncoated wires (control).

Physical Vapored Deposition with thermal evaporation (1KW SCR STACK & DRIVER model 3AM, Edwards, England) were used to deposit thin films of ZnO on SS orthodontic arch wires. In PVD, heat is produced by filaments that is thin sheet metal pieces of suitable high temperature metals such as tungsten. This extremely high temperature with vacuum chamber results in vaporized Zn. Zn travel towards the chamber and hits its substrate (SS wires). The coating process was performed by holding one side of the wire and rotating it under 250 °C for 4–5 h [22].

Coated wire specimens were incubated at 37 °C in separate vials with 5 mL Oral-B fluoride mouthwash (Procter & Gamble, Weighbridge, U K), with a pH value of 5.6 and 0.05% sodium fluoride and distilled water for 1.5 h, approximating 3 months of 1-minute daily topical fluoride treatments [19]. Then, the wire surfaces were evaluated using SEM (SEM; TESCAN – VEGA 3; TESCAN; Czech republic) by completion of the immersion period.

Corrosion of the samples was achieved via the potentiodynamic polarization technique, employing a three-electrode configuration which consisted of reference, counter, and working electrodes. The working electrode was the SS wire. The reference electrode was an Ag/AgCl electrode and the counter electrode was a platinum mesh. The purpose was measurement of the corrosion rate of Zn-coated and uncoated SS wires, which were considered as working electrode. We used two solutions which were different in pH and fluoride containing because Potentiodynamic polarization is a technique in which the electrode potential is varied at a selected rate by application of a current through the electrolyte. The corrosion rates of both coated and uncoated SS wires were assessed in neutral artificial saliva (4.1mM KH_2_PO_4,_ 4mM Na_2_HPO_4,_ 24.8mM KHCO_3,_ 16.5 mM NaCl, 0.25mM CaCl_2_ in distilled water, pH = 7.3) and the acidic one which contained Oral-B fluoride mouthwash.

The distal end of all specimens were mounted in self-cure epoxy resin to mask the electrical contacts. The exposed length of the specimen which was a 19 mm arched shape wire was cleaned with solvent and rinsed with distilled water prior to exposure. Then, electrical connections were attached between the potentiostat and the 3 electrodes with a connection pin to fix the electrode holders onto the electrode suspenders and then turned the potentiostat on. Experiments were done using IVIUM A32700 potentiostat system. For each specimen, the open-circuit potential (OCP) was recorded. During OCP measurements, the potential of the samples in the solution remained stable for a duration of 15 min.

Polarization curves were recorded at a scan rate of 1 mV/s and the potential step was 0.5mV. Corrosion parameters were extracted from the polarization curves including corrosion potential (CP) which is known as E_corr_ and corrosion current density (CD) which is also known as i_corr_. The corrosion potential and current density were calculated using Tafel curve. Experiments were done by scanning the potential from the negative to the OCP previously measured, while the final potential was positive. The potential range was ± 250 mV versus OCP at a scan rate of 1mV /s. Anodic and cathodic reactions occurred spontaneously on the electrode surface when a metallic electrode was immersed in a corrosive aqueous environment, triggering the corrosion of the electrode. Each electrochemical measurement was performed in duplicate to ensure repeatability.

The corrosion current, i_corr_, is related to the slope of the plot through the following equation:

Where &E/&I is the slope of the polarization resistance plot, β_a_ and βc are anodic and cathodic Tafel constants, and i_corr_ is corrosion current (µA/cm^2^). The corrosion current can be related directly to the corrosion rate through the following equation:

$$Corrosion Rate\left(mpy\right)={0.13i}_{corr}\times \frac{E.W.}{d}$$

where MPY is milli-inches per year, E.W. is equivalent weight of the corroding species (g) which is equivalent to the atomic weight of the corroding element divided by the valence of the element, d is the density of the corroding element (g/cm3), and i_corr_ equals the corrosion current density (µA/cm^2^) [23].

The experiments of the electrochemical impedance spectroscopy (EIS) were collected at the value of OCP with a starting frequency from 100 kHz to 100 mHz at an AC wave of ± 5 mV peak-to-peak overlaid. The electrochemical impedance data were used to predict the ohm resistance of the electrolyte (R_s_) and the charge transfer resistance (R_ct_). The impedance was expressed as the real part of the impedance (Z’), which is the resistance and imaginary part (Z”). Data analysis was performed using Zview and Ivium Soft softwares.

## Results

### Surface characterization

**Figs. 1:**
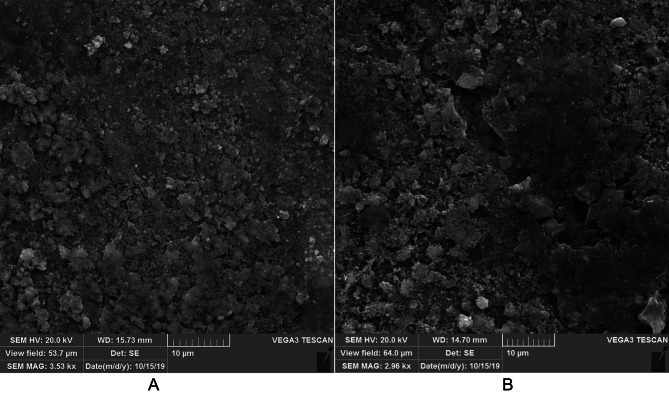
SEM photographs. Figure 1_**A**: Zn-coated SS wire and Fig. 1_**B**: Zn-coated SS wire after fluoride immersion

SEM micrographic view of Zn-coating demonstrated a semi-intact coating on SS wires. Figure 1 is the Zn-coated SS wire after 1.5 h fluoride immersion, approximating 3 months of 1-minute daily topical fluoride treatment. This time limit was introduced by Mary P. Walkera et al. [19]. . The homogeneity of Zn-coating almost remained after fluoride immersion, and the surface changes induced by fluoride agents on the SS wires were not noticeable. As the coating was found to be almost stable upon SEM evaluation after fluoride immersion, it can be concluded that adding Zn-coating may prevent the corrosion of SS from the fluoride.

### Corrosion determination

#### Tafel polarization tests

**Fig. 2:**
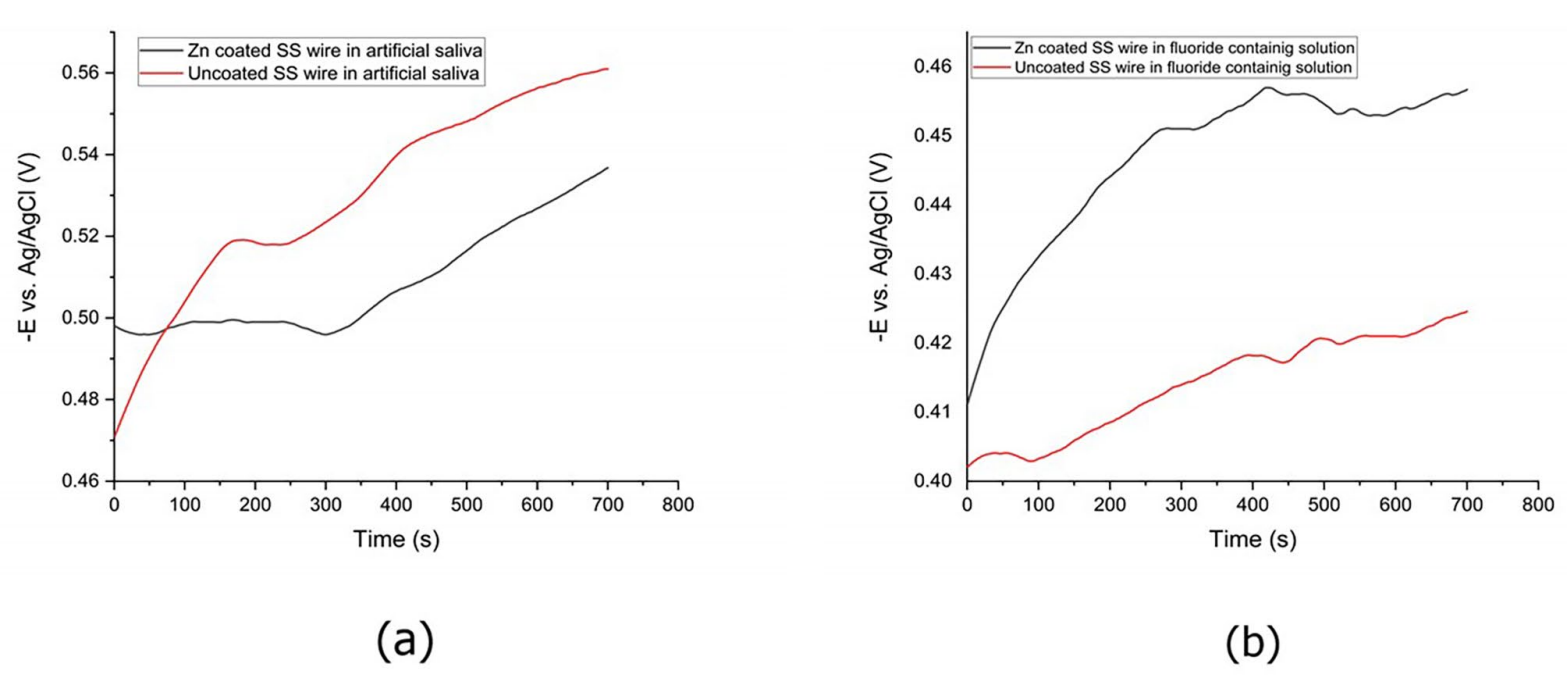
OCP diagrams of Zn-coated and uncoated SS wire in (**a**) artificial saliva and (**b**) fluoride-containing solution

**Fig. 3:**
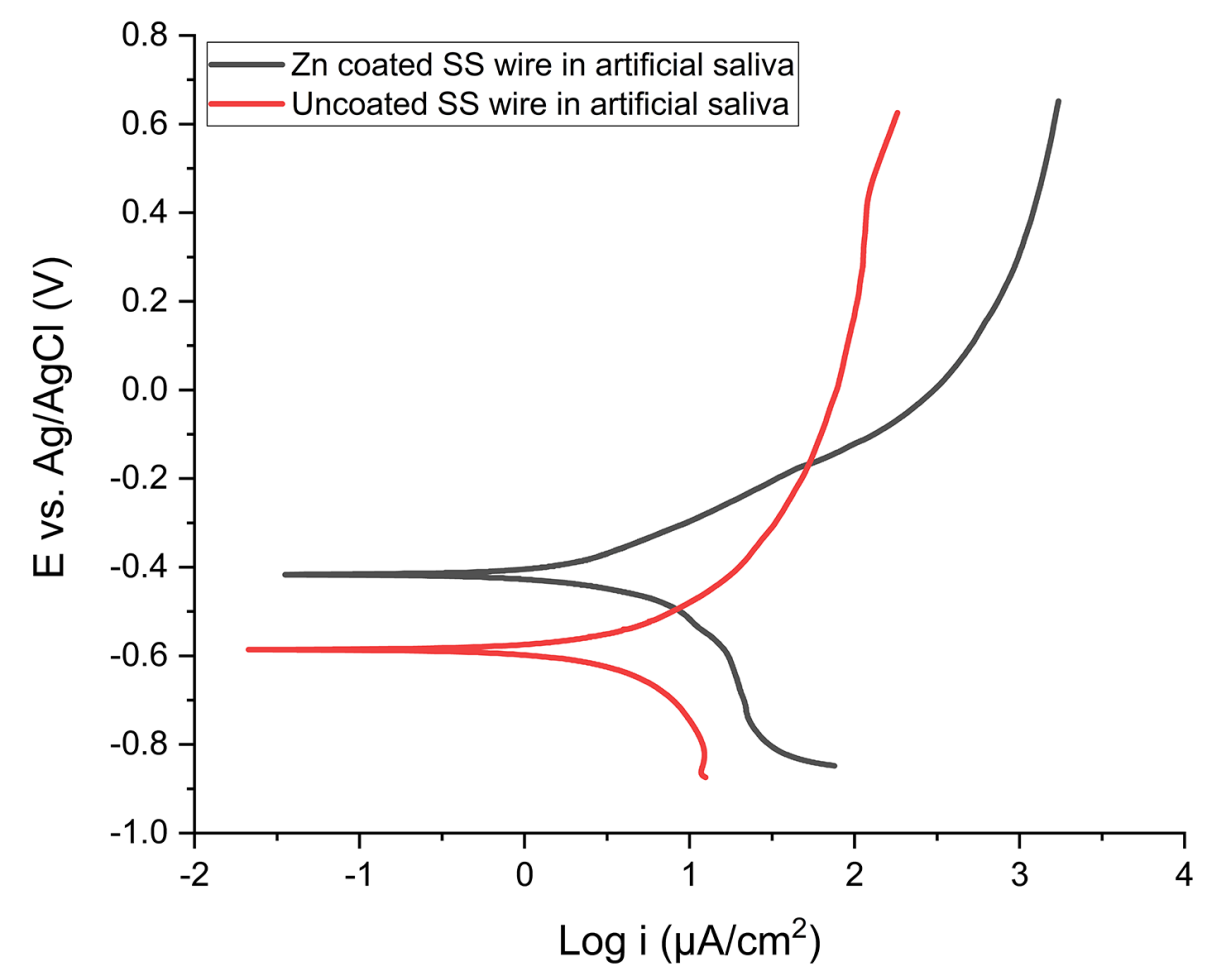
Tafel plot of Zn-coated and uncoated SS wire in artificial saliva

Potentiodynamic polarization was used to investigate the corrosion resistance of each sample. Before potentiodynamic polarization, open circuit potential (OCP) measurements were conducted. The result of OCP measurement for each specimen is shown in Fig. 2. The corrosion behaviors of Zn-coated and uncoated SS wires were compared with each other by Tafel slope in artificial saliva as an electrolyte (Fig. 3). The values of the corrosion potential (E_corr_) and the corrosion current density (i_corr_) were extracted from the curves using the Tafel extrapolation method shown in Table 1. The E_corr_ values were − 416 mV for the Zn-coated wire which was positively shifted compared to the E_corr_ of the uncoated SS wire (-584 mV).

**Table 1:** Tafel data parameters

β_c_ (mV/dec)	β_a_ (mV/dec)	E_corr_ vs. Ag/AgCl (mV)	i_corr_ (µA/cm^2^)	Sample
-127.60	100.91	-314	6.39	Uncoated SS wire in fluoride containing solution
-91.32	132.52	-391	4.69	Zn coated SS wire in fluoride containing solution
-178.38	154.85	-584	6.26	Uncoated SS wire in artificial saliva
-108.86	168.85	-416	5.20	Zn coated SS wire in artificial saliva

**Fig. 4:**
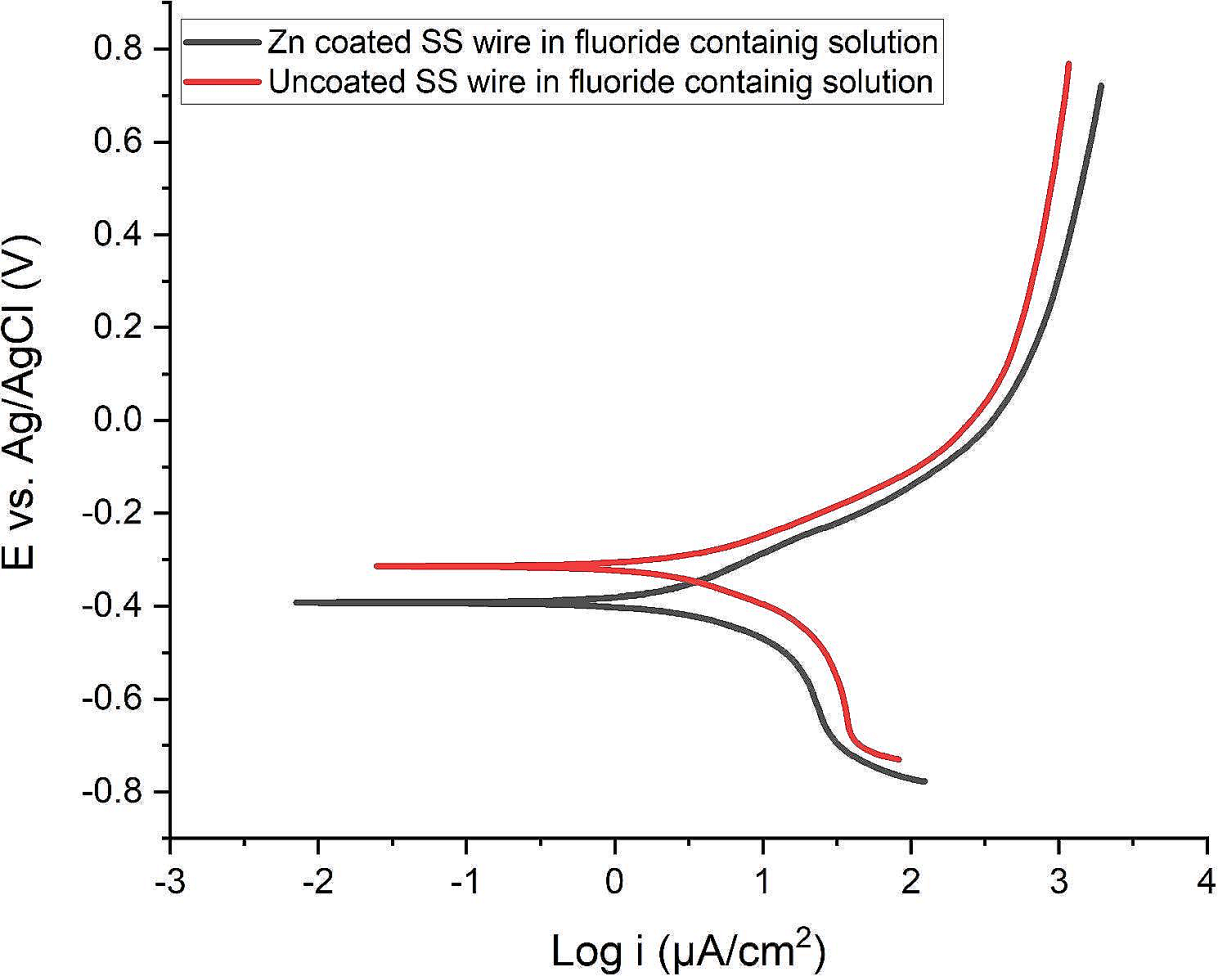
Tafel plot of Zn-coated and uncoated SS wire in the fluoride-containing solution

The i_corr_ values were 5.20 µA/cm^2^ and 6.26 µA/cm2 for the Zn-coating and uncoated SS wire, respectively. i_corr_ of the un-coated wires was higher than that of the Zn-coated ones. The corrosion parameters, E_corr_ and i_corr_, were also calculated by extrapolating Tafel curves in flouride- containing solution (Fig. 4), which was a more acidic medium than artificial saliva. The E_corr_ value of Zn-coated samples was − 391 mV, that showed a small negetive change from that of the uncoated ones (-314 mV); however, the i_corr_ of the Zn-coated SS wires was 4.69 µA/cm^2^, which demonstrated a lower value than the i_corr_ of the uncoated one(6.39 µA/cm^2^). All data are summerized in Table 1.

The calculated values of corrosion rates converted to milli-inches per year (MPY) were 2.75 for Zn-coated SS wire and 2.91 for uncoated SS wire in artificial saliva and the corrosion rate of 2.87 and 2.81 MPY for uncoated and Zn-coated SS wires, respectivly, in the flouride-contianing medium. The results showed the lower corrosion rate of the Zn-coated samples in both media.

As shown in Table 1, it was noticed that the anodic Tafel slope, βa, of Zn-coated SS wire was larger than that of the βa of the uncoated one in both artificial saliva and flouride- countianing sulotion. This could be due to presence of less active anodic sites over the surface of coated SS wires which results in the appearance of the diffusion plateau in polarization diagrams. The presence of zinc coating would polarize the sample surface and make the surface less active.

#### Electrochemical impedance spectroscopy (EIS)

**Fig. 5:**
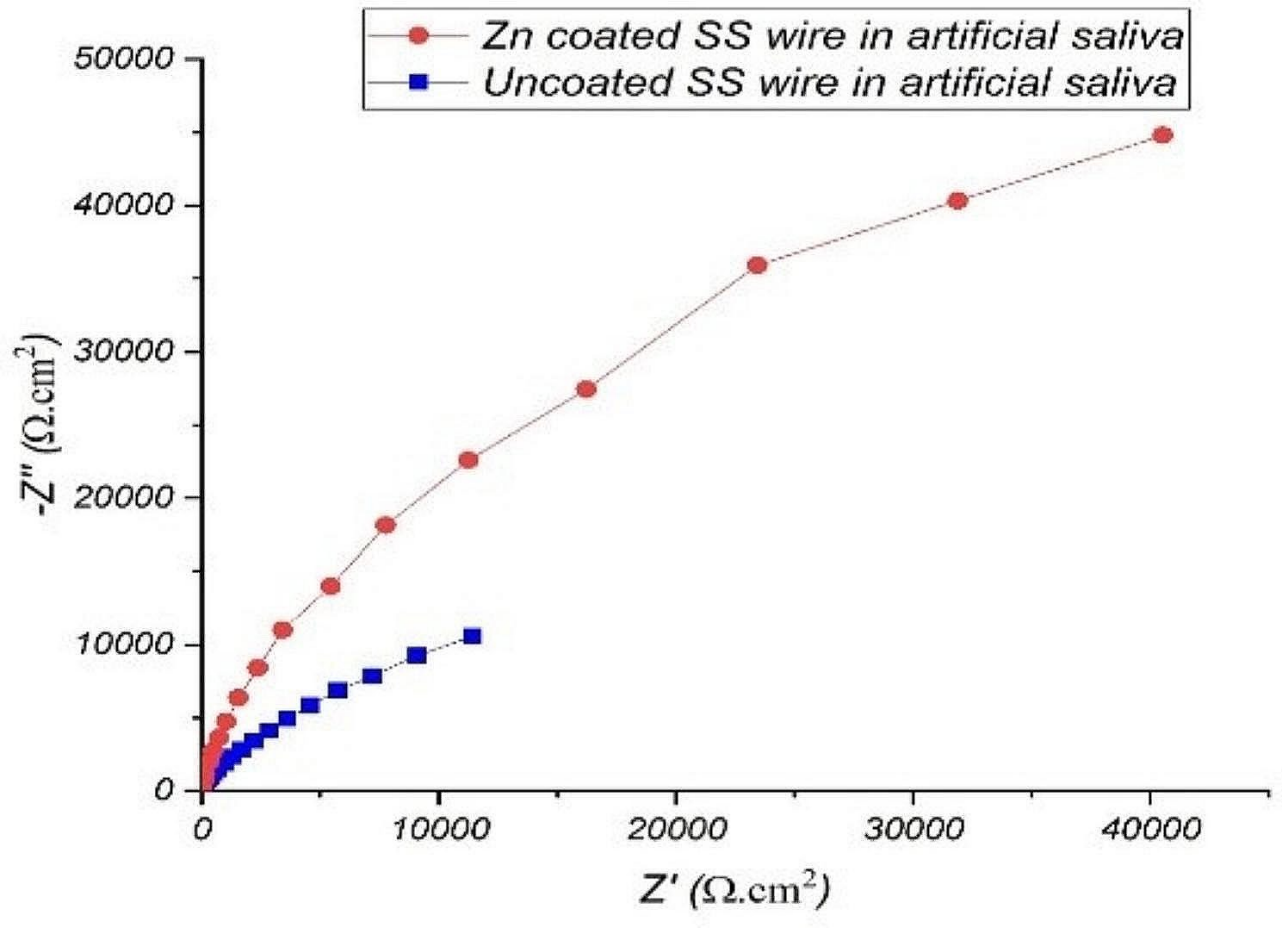
Nyquist plot of Zn-coated and uncoated SS wire in the artificial saliva

**Fig. 6:**
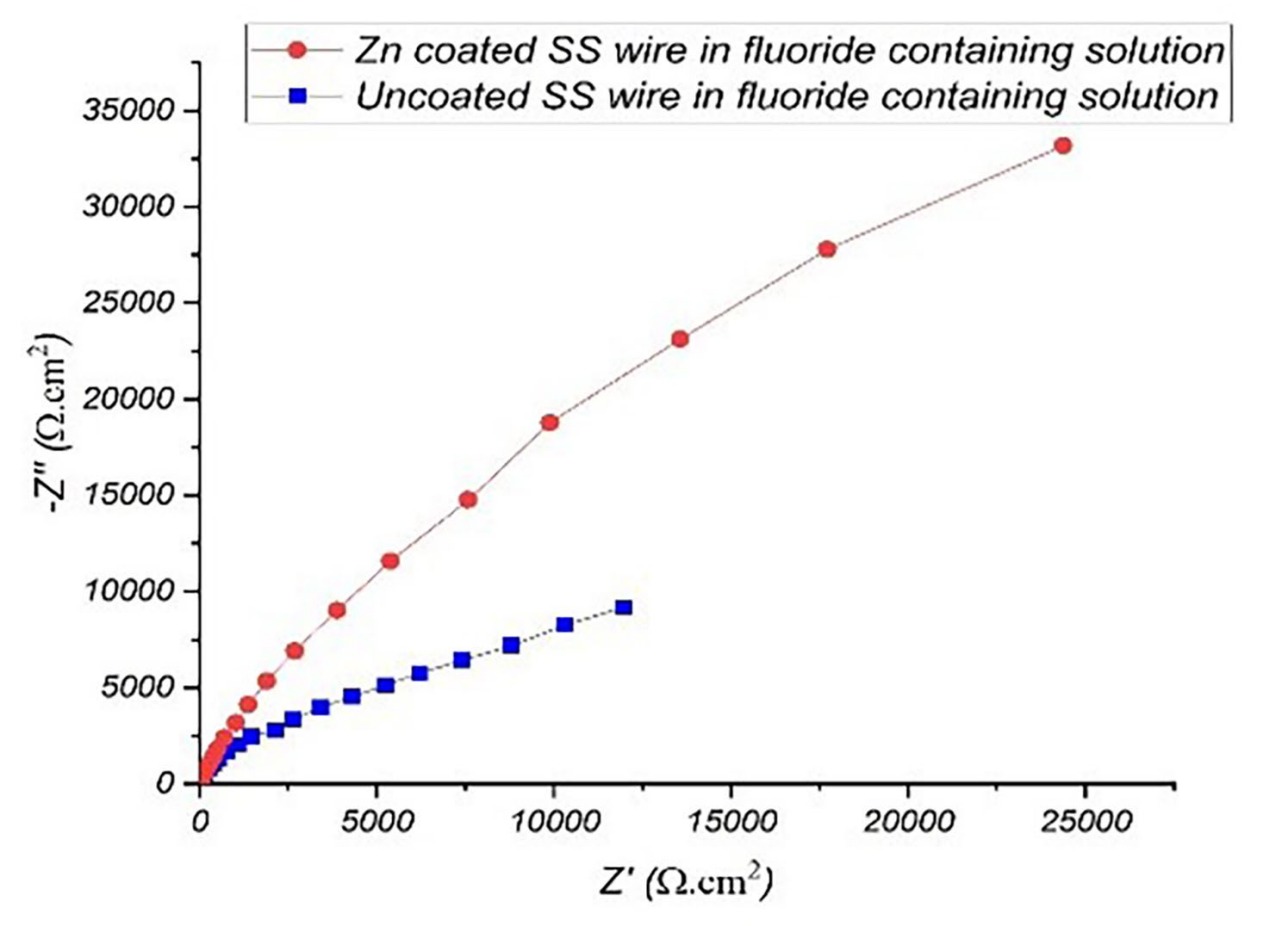
Nyquist plot of Zn-coated and uncoated SS wire in the fluoride-containing solution

**Fig. 7:**
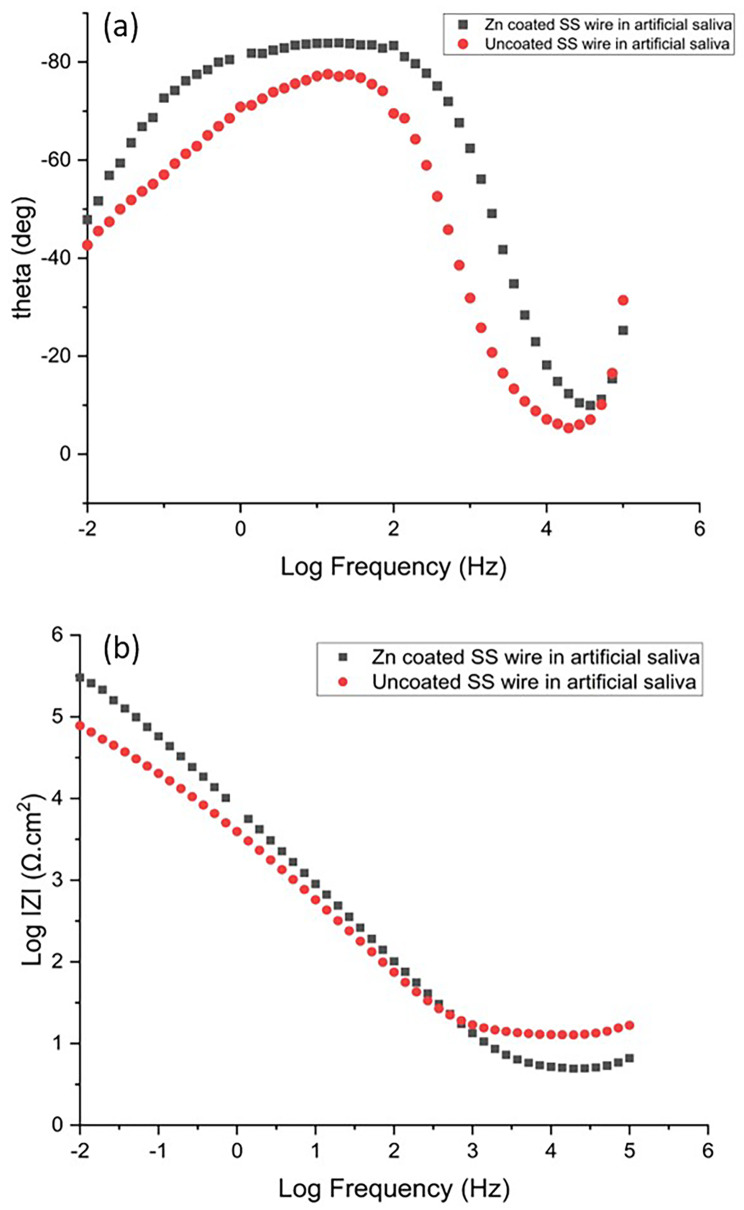
Bode plots of Zn-coated and uncoated SS wire in artificial saliva (**a**) bode phase and (**b**) bode amplitude curves

**Fig. 8:**
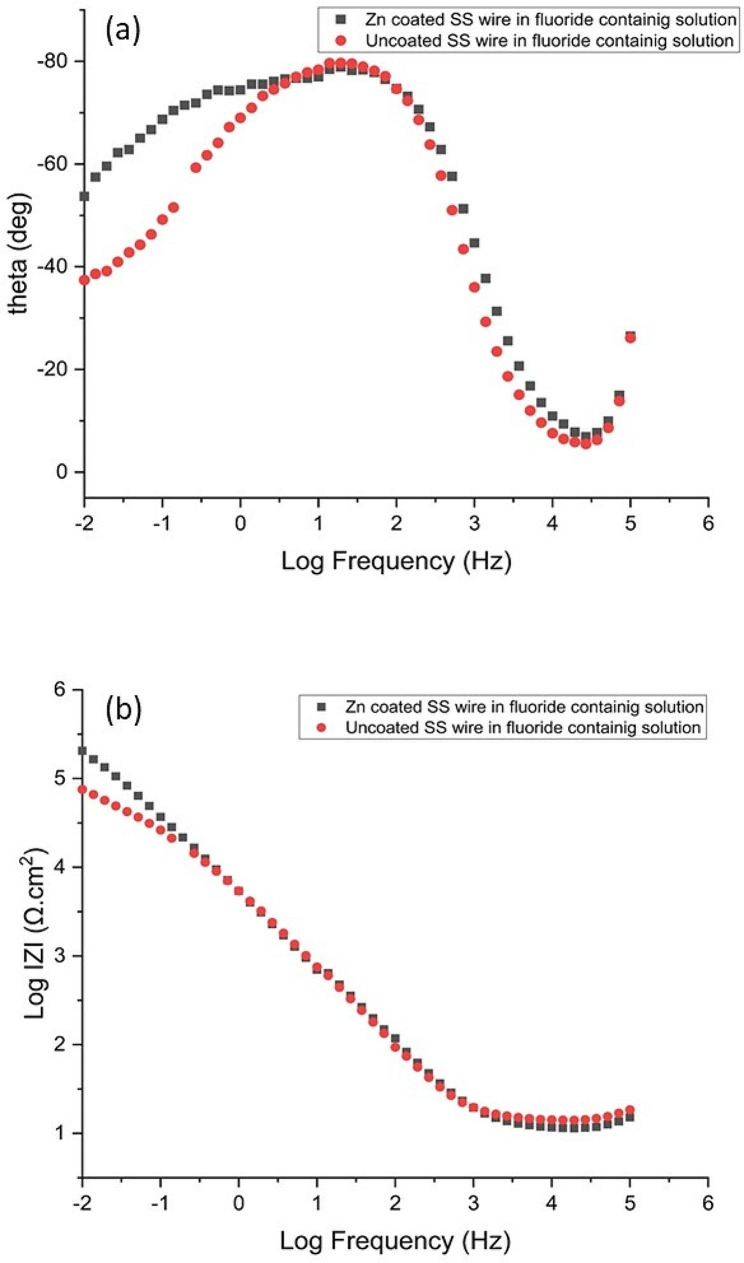
Bode plots of Zn-coated and uncoated SS wire in the fluoride-containing solution (**a**) bode phase and (**b**) bode amplitude curves

Electrochemical impedance spectroscopy was performed in order to confirm the results obtained with polarization tests. Figures 5 and 6 show the impedance Nyquist diagrams recorded for both Zn-coated and uncoated SS wires in artificial saliva and fluoride-contianing medium, respectively, in which the imaginary impedance (Z’’) is plotted against the real impedance (Z’). According to Bode plotes in Figs. 7 and 8, the uncoated and coated samples were simuleted based on the presence of one and two time constants, respectively.

**Fig. 9:**
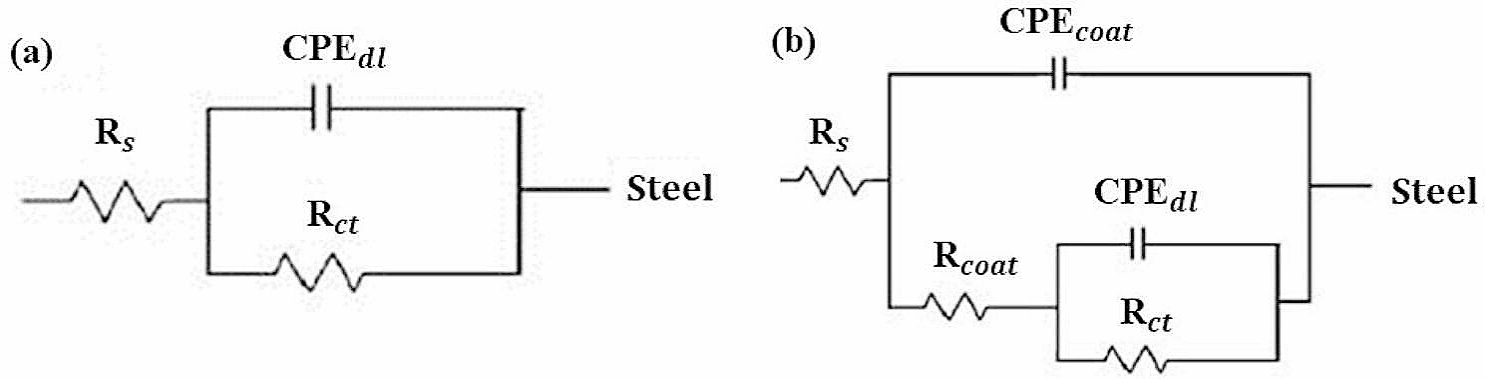
Equivalent circuits used for fitting the impedance diagrams for (**a**) uncoated and (**b**) Zn-coated samples

The impedance diagrams were fitted by the equivalent electrical circuits as shown in Fig. 9. Measurements taken at high frequencies will generally reveal the solution resistance (R_S_), while measurements taken at low frequencies will measure the charge transfer or polarization resistance(R_ct_). The numerical values of EIS data are summerized in Table 2. As it is evident from EIS parameters in Table 2, R_ct_ of Zn-coated SS wires was 552,820 and 533,140 Ω.cm^2^ in artificial saliva and fluoride contianed medium, respectively, which is higher than the R_ct_ of uncoated one. The results revealed that coated sample exhibited a better coating resistance in artificial saliva with respect to fluoride contianed medium.

**Table 2:** EIS data parameters

Sample	${R}_{s}$ (Ω. ${cm}^{2}$)	${C}_{coat}$		${R}_{coat}$ (Ω. ${cm}^{2}$)	${CPE}_{dl}$		${R}_{ct}$ (Ω. ${cm}^{2}$)	Chi-square
		${\text{Y}}_{coat}$ (Sn/ Ω.${cm}^{2}$)10 − 6	${n}_{coat}$		${\text{Y}}_{dl}$ (Sn/ Ω. ${cm}^{2}$)10 − 6	${n}_{dl}$		
Zn coated SS wire in fluoride containing solution	12.08	25.33	0.91	1920	14.89	0.71	533,140	0.02
Zn coated SS wire in artificial saliva	13.57	38	0.91	3091	36.81	0.46	552,820	0.02

The parameter R_s_ which represents the resistance of the solution occurring between the sample and the reference electrode is also listed in Table 2. Thus, the solution resistance (R_s_) values for the artificial saliva and fluoride contianed solution were about the same order.

Experimental results from Tafel plot analyses were consistent with that of electrochemical impedance spectroscopy analyses for both biological solutions. The values of n are associated with the non-uniform distribution of current as a result of roughness and surface defects. The values of the coefficient n can vary from 0.5 to 1. Higher n values are indicative of more uniform surface characteristics. Y and n are constant phase element parameters that take into consideration the formation of the protective film. Any factor that may have caused defects in the oxide film could have an impact on the constant phase element and Rct values [24].

## Discussion

In current study, we evaluated the corrosion resistance of Zn-coated SS wires. The coating was done by PVD in contrast to the study of Chang et al. who coated Zn particles by CVD [25]. The advantage of PVD coating is the thinner and more uniform layer with good mechanical properties [6].

Coating thickness is very important in orthodontic treatments because this might impede the amount of tooth movement by increasing the friction resistance between the arch wire and bracket slot. The mean Zn thickness was 0.28 ± 0.006 μm in the current study [22]; however, Krishnan et al. deposited a thin layer of TiAlN and WC/C on β-titanium orthodontic arch wires by PVD method and reported that the mean values for coating thickness obtained over substrate β-titanium arch wires were 6.56 and 1.66 μm for TiAlN and WC/C, respectively, which is significantly a thicker layer than that of our result [26].

In this study, we adopted the potentiodynamic method and electrochemical impedance spectroscopy to investigate the electrochemical corrosion resistance of Zn-coated SS wires in a artificial saliva and flouride-containing solutions. However, Kim et al. used only potentiostatic anodic polarization to determine the corrosion poteiontial of SS, NiTi, coated NiTi and titanium orthodontic wires [27]. Asachi et al. evaluated the corrosion resistance of uncoated and coated NiTi for Orthodontic Wires with Electrochemical impedance spectroscopy (EIS) and linear potentiodynamic polarization (LPP) techniques [28].

As mentioned, there was a change in E_corr_ between Zn-coated and uncoated SS wires in artificial saliva solution. The positive shift in E_corr_ can be attributed to the improved corrosion resistance of the Zn-coated samples because any increase in the corrosion potential might indicate the improvement in corrosion protection performance. Shajudheen et al. used Tafel polarization plots of ZnO thin films deposited on 304 L SS before and after salt spray test to predict the anticorrosion properties. They showed that the equilibrium E_corr_ of the ZnO films before salt spray test was positively shifted compared to that of bare stainless steel. They claimed that the shift in E_corr_ can be attributed to the improved corrosion resistance of the coated samples [16]. Also, Hosseini et al. reported the anticorrosion properties of Polypyrrole (PPy) and PPy-ZnO coating on mild steel and observed the incorporation of ZnO nanorods in the PPy coating resulted in the positive shift of E_corr_ value, indicating improved corrosion protection [29]. The current density has a strong inverse influence on corrosion resistance; higher values of corrosion current indicate less corrosion resistance or larger corrosion rate [30].

Based on the comparison between the i_corr_ of the samples in both artificial saliva and fluoride contained solution, Zn-coated SS wires exhibit better corrosion resistance against the uncoated samples because the i_corr_ of uncoated SS wires was higher than that of Zn-coated SS wires. In the same line with our study, Mareci et al. indicated a decrement in the i_corr_ of the PTFE-coated NiTi orthodontic wires with respect to the uncoated samples [28]. They demonstrated that the PTFE-coated sample had a greater corrosion resistance than the uncoated one. The steeper slope of anodic branch(βa) related to Zn-coated wires in Tafel plot indicates the decrease in anodic reaction rate in comparison with that of the cathodic one. We observed a decrease in anodic reaction of Zn-coated wires in both artificial saliva and flouride containing sulotion.

R_ct_ is the factor that determines corrosion resistance of the alloys. This value is inversely proportional to i_corr_; hence, high values of R_ct_ correspond to low values of corrosion rate [31]. We encountered better corrosion resistance of Zn-coated SS wires compared to the uncoated ones. This was probably due to the high polarization resistance which is in a good agreement with the results reported by Karuppasamy Prem Ananth et al. They used electrochemical impedance spectra in the form of Nyquist plots for the pristine 316 L SS which was coated by Mn-HAp, ZnO and Mn-HAp/ZnO bilayer coating on 316 L SS in SBF solution. By comparing the polarization resistance (R_ct_) value of those three different coatings on 316 L SS with each other, they reported that Mn-HAp/ZnO bilayer SS coated SS had more corrosion protection than other samples due to its maximum R_ct_ value(3400 Ω/cm2) [32]. To confirm the presence of fluoride-induced corrosion products, further specialized studies are recommended, such as X-ray diffraction or spectroscopic evaluation.

## Conclusion

The values of E_corr_, i_corr_, and R_ct ,_which were the electrochemical corrosion characteristics, reported better corrosion behavior of Zn-coated SS wires compared to uncoated ones in both artificial saliva and flouride containing environments. Thus, applying Zn coating on bare stainless steel orthodontics wire by PVD method might effectively increase the corrosion resistance of the underlying stainless steel substrate.

## Data Availability

The data is available on demand. Email: mansour.rahsepar@gmail.com.
